# In vitro biological and antimicrobial properties of chitosan-based bioceramic coatings on zirconium

**DOI:** 10.1038/s41598-021-94502-z

**Published:** 2021-07-23

**Authors:** Salim Levent Aktug, Salih Durdu, Selin Kalkan, Kultigin Cavusoglu, Metin Usta

**Affiliations:** 1 Materials Science and Engineering, Gebze Technical University, 41400 Gebze, Turkey; 2 Industrial Engineering, Giresun University, 28200 Giresun, Turkey; 3 Bioprocess Engineering, Giresun University, 28200 Giresun, Turkey; 4 Department of Biology, Giresun University, 28200 Giresun, Turkey; 5 Materials Institute, TUBITAK Marmara Research Center, 41470 Gebze, Turkey

**Keywords:** Biomaterials, Techniques and instrumentation, Biological techniques

## Abstract

Ca-based porous and rough bioceramic surfaces were coated onto zirconium by micro-arc oxidation (MAO). Subsequently, the MAO-coated zirconium surfaces were covered with an antimicrobial chitosan layer via the dip coating method to develop an antimicrobial, bioactive, and biocompatible composite biopolymer and bioceramic layer for implant applications. Cubic ZrO_2_, metastable Ca_0.15_Zr_0.85_O_1.85_, and Ca_3_(PO_4_)_2_ were detected on the MAO surface by powder-XRD. The existence of chitosan on the MAO-coated Zr surfaces was verified by FTIR. The micropores and thermal cracks on the bioceramic MAO surface were sealed using a chitosan coating, where the MAO surface was porous and rough. All elements such as Zr, O, Ca, P, and C were homogenously distributed across both surfaces. Moreover, both surfaces indicated hydrophobic properties. However, the contact angle of the MAO surface was lower than that of the chitosan-based MAO surface. In vitro bioactivity on both surfaces was investigated via XRD, SEM, and EDX analyses post-immersion in simulated body fluid (SBF) for 14 days. In vitro bioactivity was significantly enhanced on the chitosan-based MAO surface with respect to the MAO surface. In vitro microbial adhesions on the chitosan-based MAO surfaces were lower than the MAO surfaces for *Staphylococcus aureus* and *Escherichia coli*.

## Introduction

The amount of zirconium that exists in the body is only 1 mg in total on average, and does not have a natural biological role in the human body^1^. Zirconium can be a potential candidate for surgical implant material due to its promising properties such as low Young’s Modulus (92 GPa) and excellent biocompatibility compared to titanium and its alloys^2–4^. However, zirconium cannot directly bond to bone tissue at an early stage after implantation due to its bioinert nature^5,6^. Moreover, the microbial property of zirconium may cause postoperative infection^7–9^. It is clear that one of the major problems with the implant surfaces is microbial colonization, whereas their bioactivity and biocompatibility are improved^10–12^. Thus, zirconium has limited medical applications. In order to overcome this disadvantage, it is vital to enhance the bioactivity and antimicrobial properties via surface treatment.

Micro-arc oxidation (MAO) enhances bioactivity, biocompatibility, and corrosion resistance^2,5,13,14^. MAO that can form porous, thick, relatively rough, and firmly adherent oxide coatings on zirconium surfaces represents a promising electrochemical coating technique^2,5,15,16^. MAO, which produces bioactive and biocompatible ceramic coatings, involves anodic oxidation in aqueous electrolytes above the dielectric breakdown voltage. The short-lived micro-discharges occur locally at weak sites that are susceptible to dielectric breakdown under the high temperatures and pressures associated with the MAO process^17,18^. Eventually, porous and rough bioceramic surfaces form on metal surfaces. The properties of the MAO coatings depend on certain experimental parameters such as the substrate, the electrolyte, voltage, current, and the treatment time^19^.

Antibiotics could be presented to the implant surface to reduce the risk of postoperative infection by preventing microbial adhesion and proliferation^20,21^. However, antibiotic resistance is an important problem that requires primary clinical attention^22^. It is well known that many important pathogens, *S. aureus* being prime among them, always exhibit highly alarming levels of antibiotic resistance^23,24^. Furthermore, bacteria forming biofilms on prosthetic surfaces are resistant to antimicrobials^25–27^. Thus, instead of antibiotics, biopolymer chitosan is preferred due to its antimicrobial properties^28^.

Chitosan is a critical antimicrobial agent that has been widely investigated in recent years^29,30^. Chitosan is a natural polysaccharide obtained from deacetylation of chitin, which is found in the exoskeletons of crustaceans and insects, and in certain fungi and microorganisms^31^. This biopolymer exhibits excellent features due to its nontoxic nature, biodegradability, and promoting cell adhesion. The importance of the antimicrobial properties of chitosan can be explained by the electrostatic interaction between chitosan and microbial cells^32–34^. Chitosan is a positively charged polymer and the protonated amino group of chitosan is available to bind to the negatively charged microbial cell wall. Moreover, it disrupts mass transport across the cell wall, accelerating the death of bacteria^33,35,36^. The disruption of the bacterial membrane also leads to inhibition of the DNA-membrane complex, which has an important role in chromosome segregation, replication, transcription, or the maintenance of the physical configuration of the DNA^37,38^. Thus, natural biopolymer chitosan is proposed as an important antibacterial agent on the MAO-coated zirconium surfaces in this work.

Usually, the chitosan layer has been deposited on the MAO-coated magnesium and titanium metal surfaces in the literature, though some research has been carried out on the fabrication and investigation of properties of chitosan-based MAO surfaces on titanium and magnesium^29,39–46^. Wang et al. investigated micro-RNA-21-loaded chitosan and hyaluronic acid nanoparticles on MAO titanium surfaces^39^. Neupane et al. fabricated chitosan coating on MAO-coated Ti surfaces modified via hydrothermal treatment^40^. Fang et al. investigated the immobilization of chitosan film containing semaphorin 3A on a MAO-coated titanium surface via the silane reaction to improve MG63 osteogenic differentiation^41^. Cheng et al. studied the deposition of cefazolin sodium/chitosan composite film on MAO coatings containing Si, Ca, and Na on titanium^44^. Li et al. investigated the biological and antibacterial properties of the micro- and nanostructured hydroxyapatite/chitosan coating on titanium^29^. Micropores were sealed by the formation of chitosan on the bioceramic MAO-coated titanium and magnesium surface. However, to our knowledge there has been no previous study on the fabrication and investigation of an antimicrobial chitosan-based biopolymer structure on MAO-coated Zr surfaces to date.

In our previous work, antimicrobial Ag, Cu, and Zn-based nanolayers were produced on MAO-coated Zr surfaces, and their biological properties were investigated in detail^7–9^. In this work, natural chitosan-based MAO coatings with antimicrobial and bioactive properties were produced on Zr metal for the first time in the literature. Firstly, porous and bioactive Ca-based bioceramic surfaces were used to coat Zr metal via the MAO technique. Following the MAO, an antimicrobial chitosan layer was applied to form a uniform coating on the MAO surface. The phase structure, functional groups, surface morphologies, elemental distributions, and hydrophilic/hydrophobic properties of all coatings were analysed via XRD, FTIR, SEM, EDX-mapping, and contact angle measurements. In addition, in vitro predictions of bioactivity under bodily conditions and antimicrobial properties for gram-positive (*Staphylococcus* *aureus*) and gram-negative (*Escherichia* *coli*) bacteria of both coatings were investigated. Finally, both coatings were compared with each other in detail.

## Experimental details

### Sample preparation

Commercial pure zirconium (Zr 702) plates were used as the metal substrates for the MAO process. Initially, the substrates were cut into pieces to a size of 30 mm × 25 mm × 5 mm. Then, the substrates were ground using 400#, 800# and 1200# SiC sandpapers. Finally, they were cleaned in acetone in an ultrasonic bath dried under warm air by a heat gun.

### MAO coating production

In this study, the MAO device (MDO-100WS-100 kW), which operated from an AC (alternating current) power supply was used as preferred in our previous studies^7–9,47,48^. The Zr substrates served as an anode (working electrode) and the stainless-steel container served as a cathode in the MAO process. The MAO electrolyte consisted of 0.25 M calcium acetate and 0.06 M β-calcium glycerophosphate. The electrolyte was prepared by dissolving of all chemicals in deionized water^7–9,47,48^. The MAO treatment was carried out at 0.292 A/cm^2^ for 10 min. The electrolyte temperature was not allowed to exceed 40 °C during the MAO process via a water-cooling circulator system. After the MAO treatment, they were dried using hot air and preserved in a desiccator.

### Preparation of chitosan solution and coating

The medium molecular weight chitosan used to prepare solutions was purchased from Sigma-Aldrich, Milwaukee. According to the manufacturer (Sigma-Aldrich, Milwaukee), the molecular weight, viscosity, the degree of deacetylation and polydispersity of medium molecular weight chitosan are as 20 kDa, 200–800 cp, 75–85% and 7.3 Mw/Mn, respectively. The chitosan was dissolved in 1.0 wt% aqueous acetic acid solution with concentrations of 1.0 vol%. The solution was stirred for 1 h until the chitosan was completely dissolved at room temperature. The MAO samples were dipped into the chitosan solution for 5 min. The then chitosan-coated MAO samples were drawn out at a constant rate and were dried at 37 °C. This procedure was repeated three times to ensure the solution covered both the micropores and thermal cracks in the MAO coating. Finally, to prevent thermal stresses, the chitosan-coated MAO samples were dried in atmosphere at room temperature for 24 h^45^. Thus, free chitosan used without rinsing might be the source of the antibacterial after dipping process.

### Surface characterization

The phase structures of the MAO coating surfaces were identified using a powder XRD device (XRD: Bruker D8 Advance) with Cu-Kα radiation at a scanning speed of 1° min^−1^ between 20° and 80°. The chitosan-based MAO coating was probed by using ATR FT IR device (FT IR: JASCO FT/IR 6600) in the wavenumber range from 4000 to 400 cm^−1^. The surface morphologies of both surfaces were determined via SEM (SEM: Hitachi SU1510). The EDX attached to the SEM was used to analyse elemental composition and amounts in both surfaces. The average contact angles were determined using a contact angle goniometer (CAG: Dataphysics OCA 15EC). The CAG device was used with a sessile drop technique through all analyses. The average contact angle measurements were carried out within 60 s using the SCA software after touching a 1 μL drop of distilled water onto both coating surfaces.

### Bioactivity properties

In vitro predictions of bioactivity for both coatings were evaluated by immersion test in simulated body fluid (SBF). For this experiment, Kokubo and Takadama's SBF recipe (1.0 × SBF) was used^49^. Both coatings were immersed for 14 days at 36.5 °C, with the SBF being refreshed every two days. The SBF was prepared by dissolving reagent-grade NaCl, NaHCO_3_, KCl, K_2_HPO_4_·3H_2_O, MgCl_2_·6H_2_O, CaCl_2_, and Na_2_SO_4_ into deionized water and buffering to pH 7.40 with (CH_2_OH)_3_CNH_2_ and 1.0 M HCl at 36.5 °C. The surface area ratio of the coating surfaces with respect to the SBF volume was set to be approximately equal to 10^49^. Both coatings were gently washed in distilled water post-immersion in SBF. Finally, they were allowed to dry at room temperature and then transferred into desiccators.

Post-immersion in SBF, both coatings were analysed via XRD, SEM, EDX-mapping, and EDX-area. The phase structures of each of the immersed surfaces were investigated via XRD (GNR Europe 600) with Cu-Kα radiation at a scanning speed of 1° min^−1^ from 20° to 80°. The surface morphology of each of the immersed surfaces were analysed via SEM (Hitachi SU1510) up to magnification of 10,000×. The elemental distribution and elemental amount on each of the immersed surfaces were investigated via EDX-mapping and EDX-area analysis.

### Antimicrobial properties

The antimicrobial properties of the uncoated and chitosan-coated surfaces were determined via agar diffusion test. The antimicrobial activity of the surfaces was tested against a gram-negative bacterium such as *Escherichia* *coli* ATCC 11293 and a gram-positive bacterium such as *Staphylococcus* *aureus* ATCC 6538. For this purpose, fresh bacterial broth was prepared from stock cultures. The bacterial cultures to be used in the agar diffusion test were prepared from the fresh medium of each strain according to McFarland 1.0 standards (10^9^ CFU). 100 µL of the prepared suspension was homogeneously spread over the surface of Müller Hinton Agar. The MAO and chitosan-coated MAO surfaces were placed in Petri dishes and incubated at 37 °C for 24 h. The diameters of the inhibition zones (mm) formed around the coating were evaluated for antimicrobial properties. Tetracycline and ceftazidime were used as positive controls for *E. coli* and *S. aureus*, respectively, and all tests were repeated in triplicate.

### Statistical analysis

Statistical analysis was performed using the “IBM SPSS Statistics 22 SP” program suite. Data were reported as mean ± SD (standard deviation). The statistical significance between the means was determined via one-way ANOVA and Duncan’s test, with *p* < 0.05 considered statistically significant.

## Results and discussion

**Figure 1:**
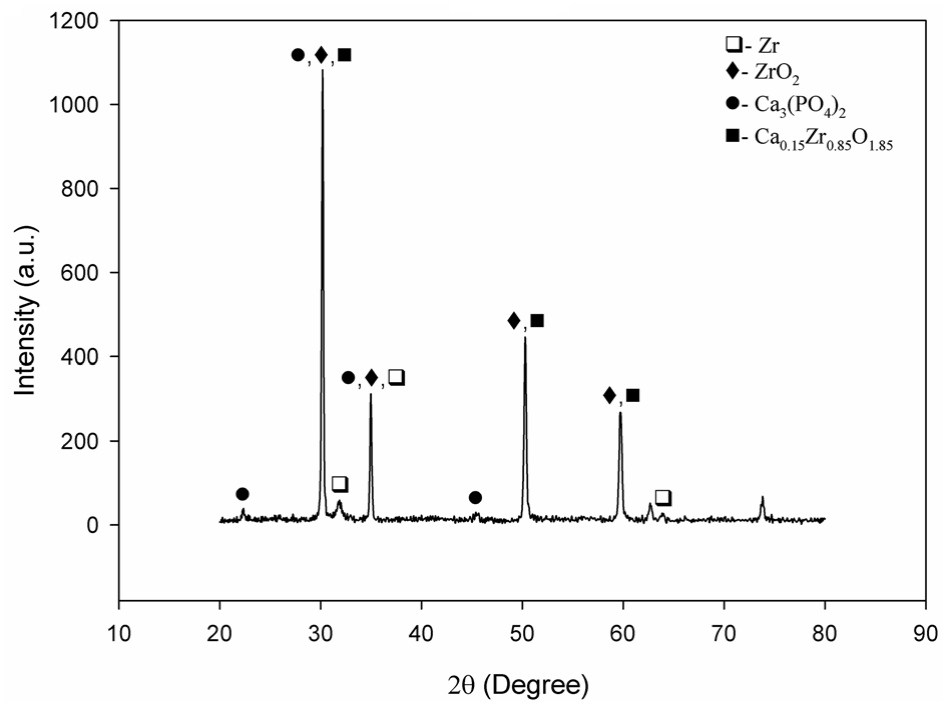
XRD spectra of the MAO coating.

The phase structure of the MAO coating was investigated via powder XRD analyses, as shown in Fig. 1. As seen in the resultant XRD spectra, the phases of Zr, cubic ZrO_2_, metastable Ca_0.15_Zr_0.85_O_1.85_, and Ca_3_(PO_4_)_2_ were detected on the MAO surface. Cubic ZrO_2_ and Ca_3_(PO_4_)_2_ were observed as the major phases, while Zr was found as a minor phase in the coating structure. The Zr signal in the XRD spectra derives from the substrate and metallic compounds on the coating. Firstly, the ZrO_2_ was formed by the reaction of oppositely charged Zr^4+^ and OH^−^ ions at high pressure and temperature due to the micro-discharge channels present in the initial steps of MAO. The instant localized temperature in the micro-discharge channels can reach up to 2500 K, as reported in the literature^50^. Therefore, stable cubic ZrO_2_ was observed throughout the entire surface. Moreover, the ZrO_2_ phase, which serves as nucleation sites, contributed to the formation of Ca-based phases such as Ca_3_(PO_4_)_2_, Ca_0.15_Zr_0.85_O_1.85_, and Ca_10_(PO_4_)_6_(OH)_2_^47,48^. Positively charged Ca^2+^ and negatively charged PO_4_^3−^ ions derived from the electrolyte reacted with each other on the ZrO_2_–based micro-discharge channels. Ca_3_(PO_4_)_2_ was then formed on the MAO surface. Simultaneously, the Zr^4+^ from the substrate and the Ca^2+^ and OH^−^ from the electrolyte combined with each other on the micro-discharge channels, subsequently forming metastable Ca_0.15_Zr_0.85_O_1.85_^51^.

**Figure 2:**
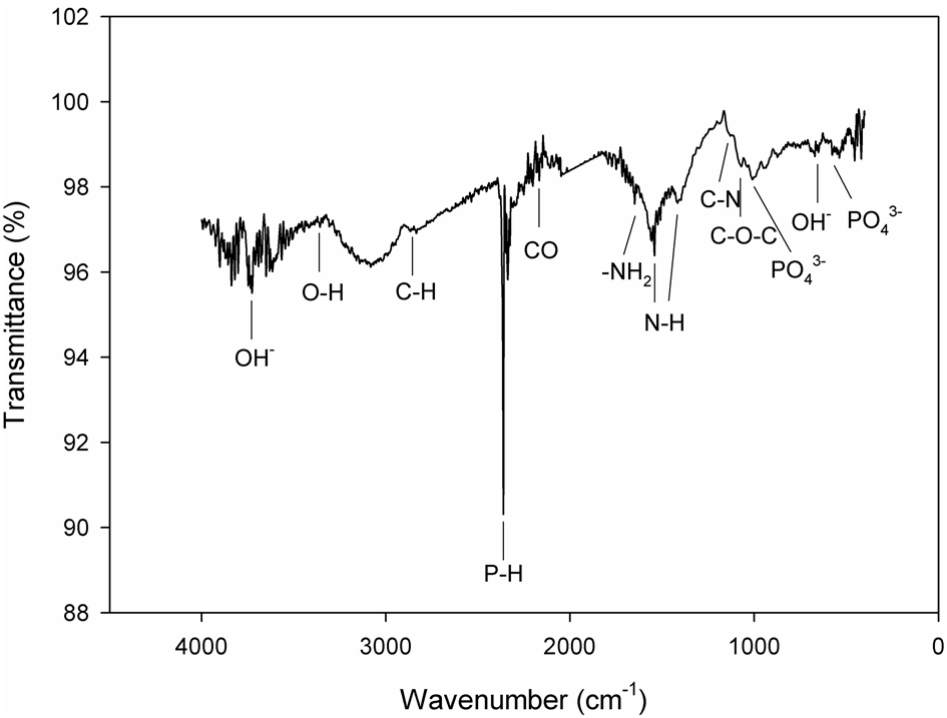
FTIR spectra of the chitosan-based MAO coating.

The FT-IR spectra of the chitosan-based MAO coatings are shown in Fig. 2, where the characteristic bands of chitosan, ZrO_2_, and calcium apatite-based structures can be observed. The FTIR peaks located at 560–570, 645–655, 1028, 1089, 1150, 1425, 1590, 1657, 2140–2165, 2340–2380, 2872, 3360–3370, and 3730–3750 cm^−1^ correspond to various vibrational modes characteristically associated with PO_4_^3−^, OH–, PO_4_^3−^, C–O–C, C–N, N–H, N–H, –NH_2_, CO, P–H, C–H, O–H, and OH^−^ species, respectively^46,51–59^. Two peaks located at 1089 and 1150 cm^−1^ are the characteristic absorption peaks of the C–O–C and C–N stretching modes, respectively^46,57^. The absorption band peaks at 1425 and 1590 cm^−1^ correspond to the N–H band^46,56,57^. The stretching peak at 1657 cm^−1^ corresponds to –NH_2_^46^. The stretching vibration band peaks at 2872 cm^−1^ can be attributed to the C–H in methyl or methenyl functional groups^46^. The stretching vibrations of non-associated peaks at 3360–3370 cm^−1^ correspond to an O–H band^58^. All of these peaks verify the existence of a chitosan-based layer structure on the MAO surface^46^. Furthermore, the other peaks support the presence of cubic ZrO_2_, Ca_3_(PO_4_)_2_, and apatite. The characteristic band peak at 1028 cm^−1^ verifies the existence of Ca_3_(PO_4_)_2_^59^. The absorption band peak at 2140–2165 cm^−1^ verifies the existence of c-ZrO_2_^51^. The stretching vibration, libration-deformation, stretching vibration, and stretching vibration band peaks at 560–570, 645–655, 2340–2380 and 3730–3750 cm^−1^ are characteristic of the existence of apatite^51–55^. However, crystalline apatite was not observed on the MAO surfaces by XRD, as can be seen in Fig. 1. Thus, it could be concluded that the MAO coatings contained an amorphous apatite structure because it could not be kinetically transformed to the crystalline form during the MAO process.

**Figure 3:**
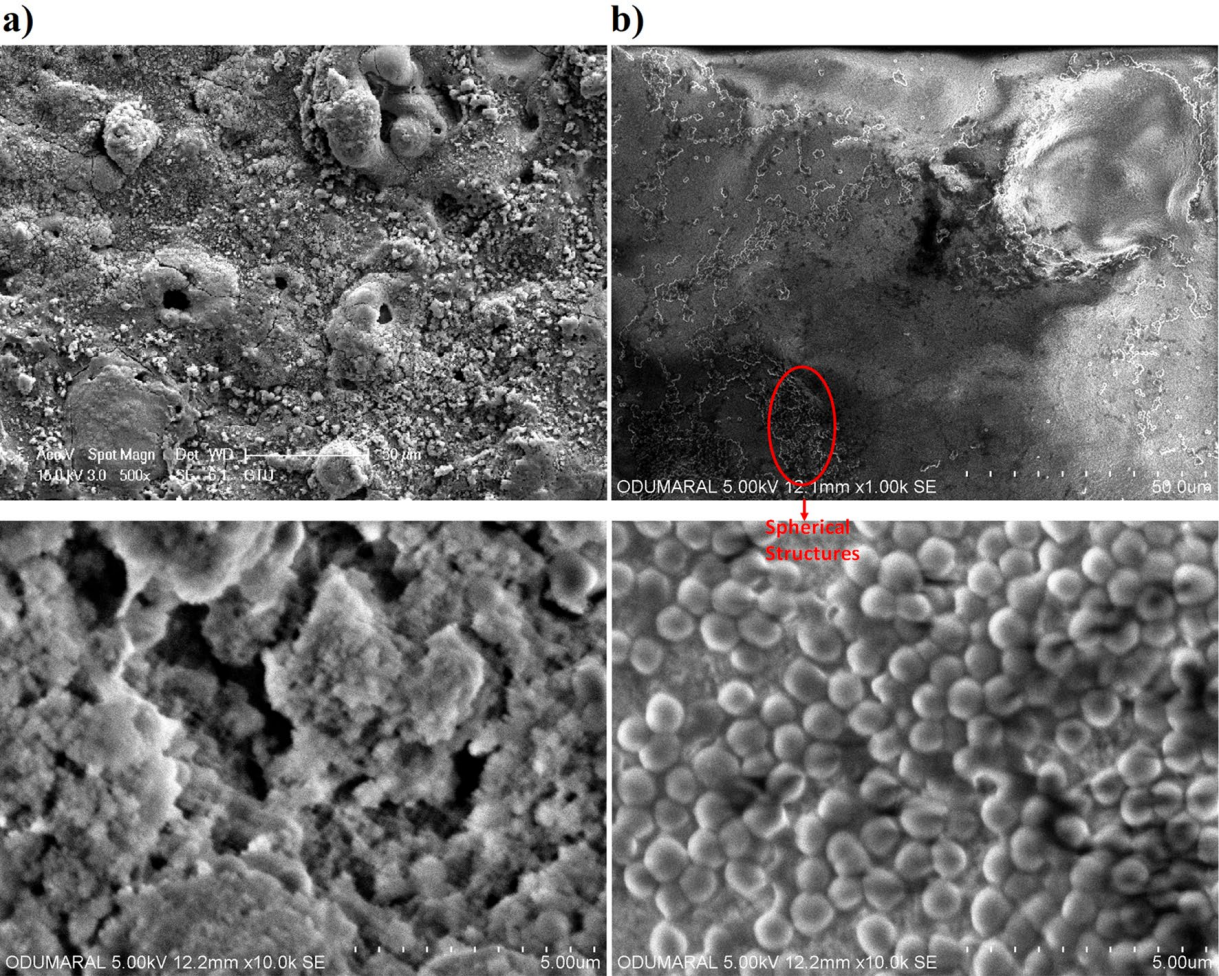
SEM images of the coatings with low and high magnifications: (**a**) the MAO and (**b**) chitosan-based MAO surfaces.

The surface morphologies of the MAO and chitosan-based MAO coatings were investigated via SEM, as can be seen in Fig. 3. The surface of the MAO coatings was highly porous and rough owing to the presence of micro-sparks during the MAO process. A large number of micropores and voids were found on the MAO surface. Cracks were found on the MAO surface because the thermal stresses between the highly localized hot surface and cold electrolyte during the process. It is well known that these types of porous and rough bioceramic surfaces are beneficial to cell attachment, proliferation, and tissue growth under bodily conditions for biomedical implant applications. All pores and voids were filled with the antimicrobial type of chitosan polymer structure when the MAO surface was dip coated, after which the homogeneous antimicrobial chitosan-based MAO surfaces were fabricated on zirconium. After being coated with a chitosan layer on the MAO surface, any micropores, voids and thermal cracks were not observed as shown in Fig. 3b. This suggests that chitosan-coated MAO surfaces were completely covered. The newly formed chitosan-based layered were observed on the surface. Spherical structures were locally observed on the MAO surface at low magnifications. It is well known that chitosan precipitates at pH 7.4^60^. A new layer formed on the MAO surface is generally non-spherical as seen in Fig. 3b.

**Figure 4:**
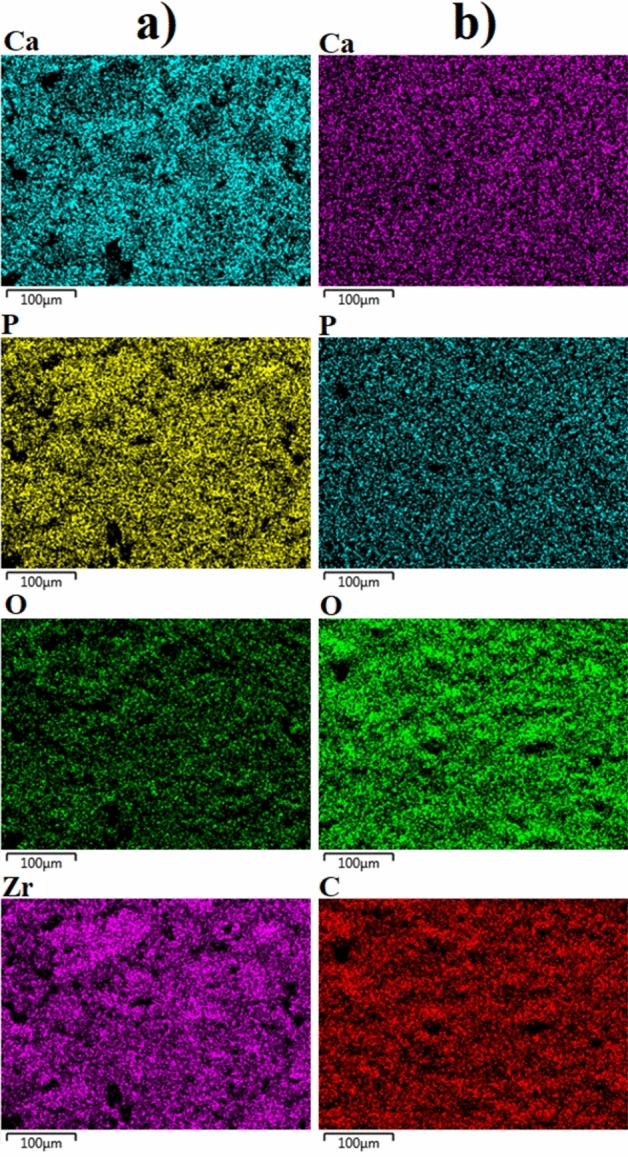
EDX-mapping analysis images of the coatings: (**a**) the MAO and (**b**) chitosan-based MAO surfaces.

The elemental distribution on both surfaces were analysed via EDX-mapping, as illustrated in Fig. 4. The elemental analysis for each surface are given in Table 1. As expected, only Ca, P, O, and Zr were detected on the MAO surface. The Ca, P, and O originated from the calcium acetate- and calcium glycerophosphate-based electrolyte, with Zr coming from the metallic substrate, as expected. Furthermore, all detected elements were homogenously distributed across the entire MAO surface, as shown in Fig. 4a. Besides the existence of Ca, P, and O, C was detected on the chitosan-based MAO surface. This element was uniformly dispersed across the surface on the post-coating chitosan layer. The chitosan structures naturally contain C and O; however, no Zr was observed on the chitosan-based MAO surface. It was concluded that the Zr-based oxide structures found on the inner layer and the outer surface mainly consisted of Ca-based bioactive and biocompatible elements and phase structures. This clearly supports the premise of the contribution of ZrO_2_ to the formation of Ca-based structures.

**Table 1:** EDS spectra results of the MAO and chitosan-based MAO coatings.

Elements	MAO coating		Chitosan-based MAO coating	
	wt%	at%	wt%	at%
Zr	23.72	6.81	–	–
O	42.14	69.04	49.03	46.36
Ca	24.68	16.14	8.09	3.05
P	9.46	8.00	4.29	2.09
C	–	–	38.45	48.42

**Figure 5:**
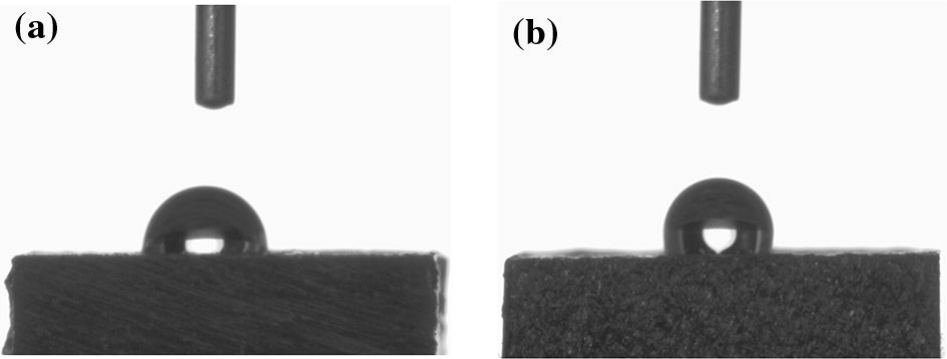
The representative images of droplets contacted on the coatings for 60 s (**a**) the MAO and (**b**) chitosan-based MAO surfaces.

The wettability of both surfaces was investigated by a sessile drop-contact angle measurement technique, as shown in Fig. 5. The average contact angles of the MAO and chitosan-based MAO surfaces were measured as 94.0° ± 0.3 and 113.5° ± 0.2, respectively. All measurements were repeated three times to get an average value of the wettability of the surfaces. Both surfaces had hydrophobic properties since the average contact angle values were greater than 90°. However, in terms of compassion, the chitosan-based MAO surface indicated the hydrophobic character of the MAO surface. Wettability mainly depends on the morphological structures/chemical composition of a given surface. The MAO surfaces, which had large numbers of voids and thermal cracks, were porous structures, as can be observed in Fig. 3a. The MAO surfaces usually exhibit hydrophilic properties owing to the capillary effect on the liquid due to the pores^61^. Thus, the water droplet on the MAO surface will be easily absorbed and spread compared to the more homogenous chitosan-based biopolymer surface. The highest initial contact angle was in agreement for the chitosan-coated substrate to the value reported in the literature, which can be attributed to the basis of its chemical properties^62^. The large initial contact angle observed might indicate the reorganization of the molecule which is presumably associated with the methyl moieties of the residual acetyl groups along the polysaccharide backbone^63^. Therefore, the wettability of the chitosan-based MAO surface was lower than that of the MAO surface.

**Figure 6:**
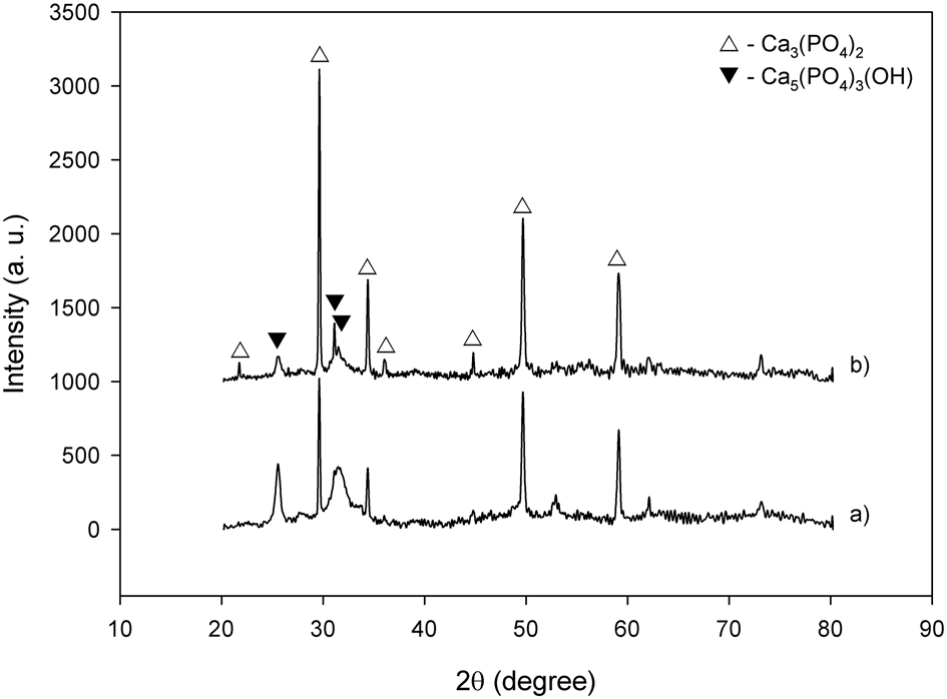
XRD spectra of the coatings at post-immersion in SBF: (**a**) the MAO and (**b**) chitosan-based MAO surfaces.

**Figure 7:**
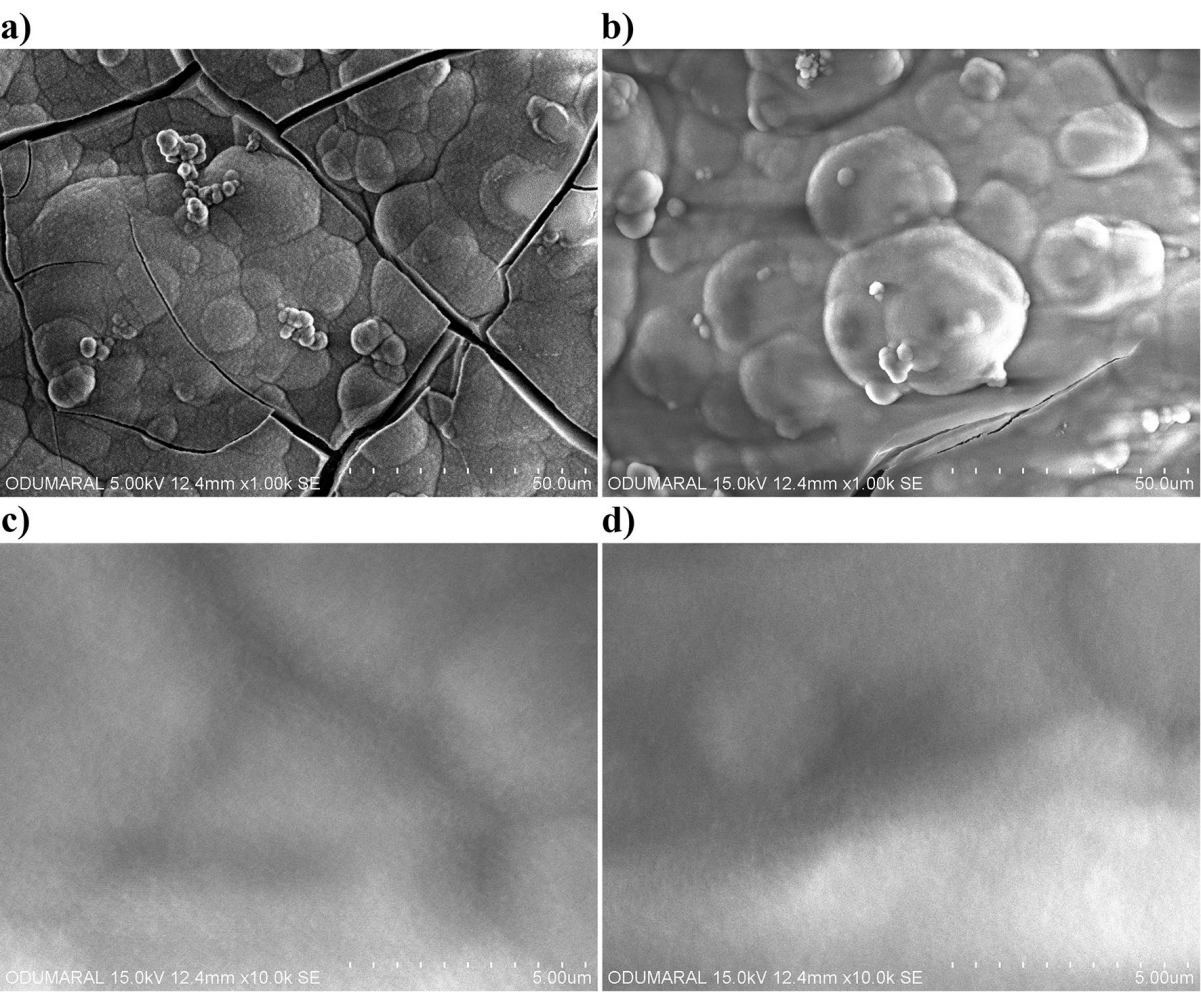
The surface morphologies of the coatings immersed in SBF for 14 days: (**a**,**c**) for the MAO and (**b**,**d**) for chitosan-based MAO surfaces.

**Figure 8:**
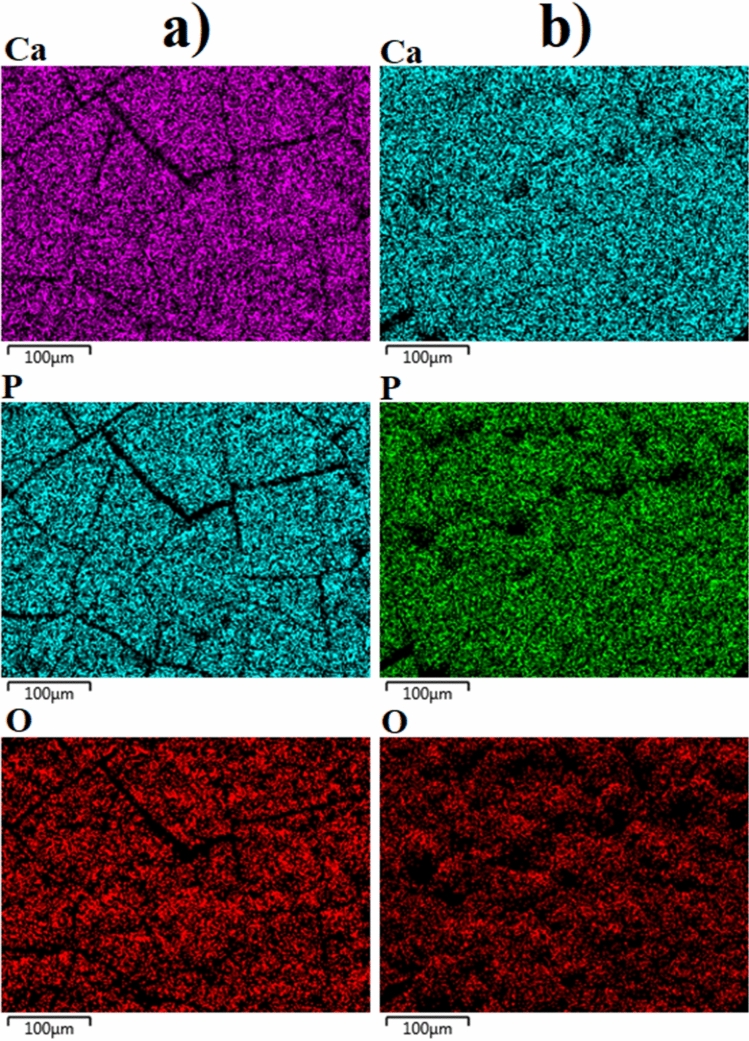
EDX-mapping analysis images of the coatings at post-immersion in SBF: (**a**) the MAO and (**b**) chitosan-based MAO surfaces.

In vitro immersion tests of the MAO and chitosan-based MAO surfaces were carried out at 36.5 °C in SBF for 14 days. It is well known that this test gives information that allows for the prediction of surface bioactivity. Post-immersion in SBF, the phase structure, surface morphology, and elemental distribution of each surface were analysed via XRD (Fig. 6), SEM (Fig. 7), and EDX-mapping (Fig. 8). Moreover, the amount of each element that had formed on each of the surfaces post-immersion in SBF is given in Table 2. As seen in Fig. 6, a TCP (Ca_3_(PO_4_)_2_) and a hydroxyapatite (Ca_5_(PO_4_)_3_(OH)) structure were detected as major phases on both surfaces. The Ca^2+^ ions, which were released from the proteins, adsorb PO_4_^3−^ ions by electrostatic interactions in the SBF solution^64^. They then simultaneously react with each other to form Ca_3_(PO_4_)_2_ during the early stages of immersion in SBF. Finally, they react with OH^−^ ions and transform to Ca_5_(PO_4_)_3_(OH) through the immersion process. The mechanism by which the hydroxyapatite structure formed on different surface types such as undoped and antimicrobial Ag-, Cu-, and Zn-doped MAO surfaces post-immersion in SBF have been discussed in detail in our previous studies^7–9,47,48^. The SBF immersion test revealed that the chitosan layer was favourable for hydroxyapatite formation. The bioactivity of chitosan originated due to the large number of protonated amino groups on the chitosan surface. Chitosan’s surface can absorb OH^−^ ions in SBF via hydrogen bonding and electrostatic attraction. Eventually, they would be adsorbed by the Ca^2+^ and PO_4_^3−^ in solution via electrostatic attraction. Finally, their reaction under SBF conditions forms the bone-like apatite on the chitosan-based MAO surface^29^. Furthermore, the chitosan layer contributes to the nucleation of hydroxyapatite because it contains a large amount of OH^−^^65^. The original porous bioceramic MAO and nonporous biopolymeric chitosan-based MAO surfaces were filled with a new secondary apatite layer post immersion in SBF. As shown in Fig. 7, the new homogeneous apatite layer formed was deposit across the entirety of both surface layers. There are some cracks in Fig. 7a with respect to Fig. 7b. The top layers of both surfaces are dissolved during immersion in SBF. Thus, micro-cracks are observed on new apatite layers on both surfaces post-immersion. The dissolved layer on the surfaces may present micro-cracks in the coatings or may propagate micro-cracks on the surface as part of the post-immersion process due to the presence of residual stresses being released^66^. However, the micro-cracks that occurred on the chitosan-based MAO surface were smaller and thinner than those on the plain MAO surface. The Ca, P, and O are necessary basic elements for the formation of apatite. Only, Ca, P, and O were observed on each surface post immersion in SBF. Furthermore, they were uniformly distributed across the whole surface, as shown in Fig. 8. Furthermore, the amount of each element of each of the surfaces post-immersion in SBF were the similar to the values reported in Table 2. As shown in Table 2, the amounts of each element, namely Ca, P, and O, detected on each of the surfaces were nearly identical. Therefore, no negative effect on the bioactivity of the chitosan-layer on the MAO surface. Moreover, these results confirmed that both coatings showed excellent bioactivity.

**Table 2:** EDS spectra results of the MAO and chitosan-based MAO coatings at post-immersion in SBF.

Elements	MAO coating		Chitosan-based MAO coating	
	wt%	at%	wt%	at%
Ca	33.23	18.62	33.32	18.69
P	18.19	13.19	18.18	13.19
O	48.57	68.19	48.49	68.12

**Figure 9:**
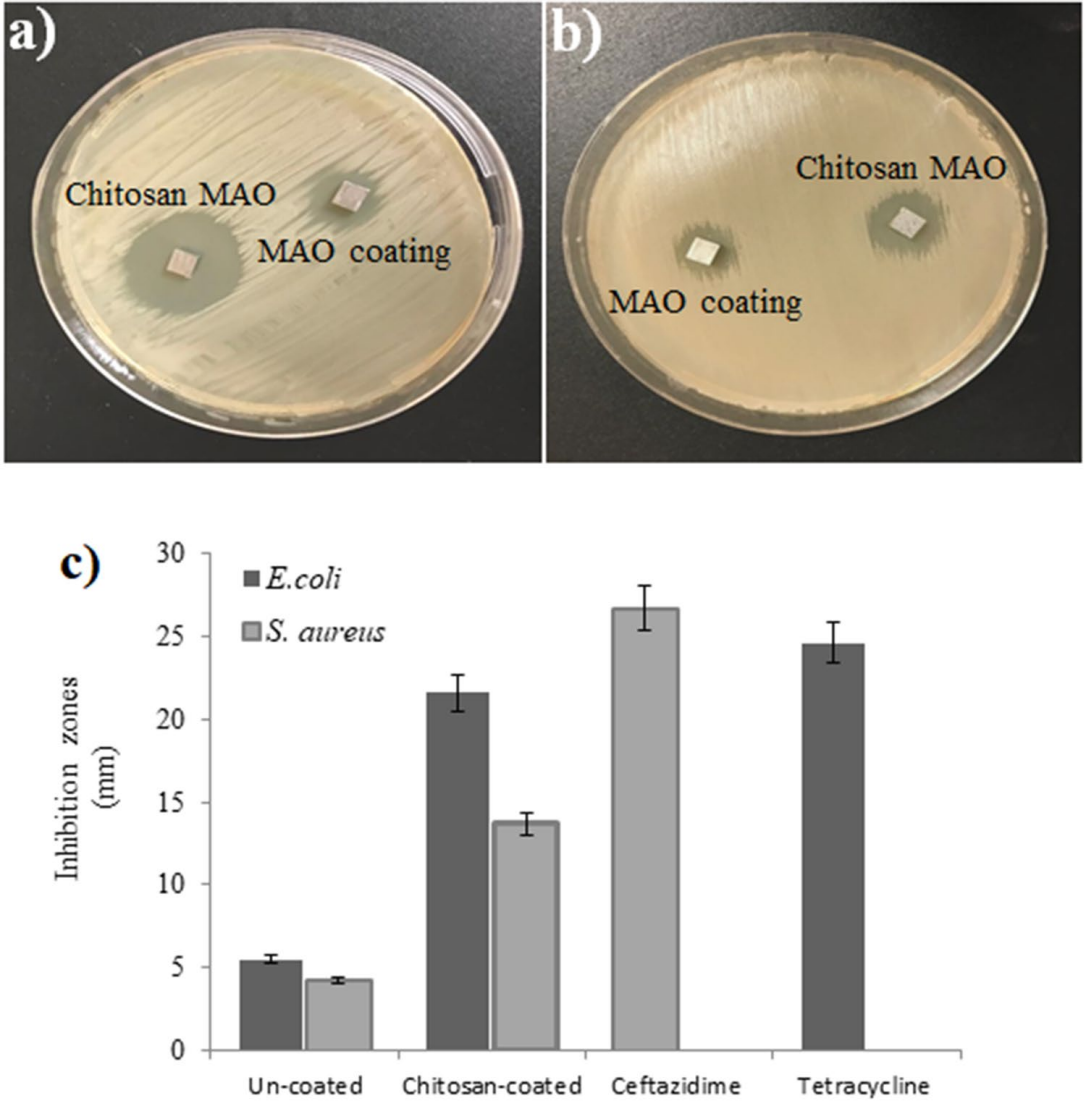
Inhibition zones of the MAO and chitosan-based MAO coated surfaces (**a**) *E. coli*, and (**b**) *S. aureus* and (**c**) inhibition zones (mm) of the MAO and chitosan-based MAO surfaces.

The antimicrobial activities of MAO and chitosan-coated MAO surfaces were examined via the agar diffusion test, the results of which are given in Fig. 9a–c. The minimum inhibition zone for both strains was obtained with the MAO surfaces. The MAO surface exhibited 5.5 ± 0.7 and 4.2 ± 0.3 mm inhibition zones against *E. coli* and *S. aureus*, respectively. It was observed that after the chitosan coating of the surface, the inhibition zones obtained against bacteria increased significantly (*p* < 0.05). Chitosan-based MAO surfaces exhibited 21.6 ± 1.3 and 13.7 ± 0.9 mm inhibition zones against *E. coli* and *S. aureus*, respectively. It was determined that chitosan-based MAO surfaces have 74.5% greater antimicrobial activity against *E. coli* than MAO surfaces. For *S. aureus*, chitosan-based MAO surfaces exhibited 69.3% more antimicrobial activity than the MAO counterparts. This result can be related to the antimicrobial properties of the chitosan coating. Free chitosan used without rinsed might be the source of the antibacterial after dipping process. Chitosan is the deacetylation product of the chitin molecule. Chitin is a linear biopolymer formed by the bonding of *N*-acetyl d-glucosamine units via glycosidic bonds^67^. Chitin is insoluble in many solvents due to its compact structure. The lack of solubility in dilute acid or alkaline solvents, and especially in water, limits chitin’s usability^68^. In order to increase its solubility and usability, chitin is subjected to deacetylation with NaOH from which high solubility chitosan is formed. Chitosan is a straight-chain polymer consisting of d-glucosamine and *N*-acetyl d-glucosamine^69^. It contains a large number of amine groups and is thus easily soluble in acidic solutions. Chitosan, which is physically, chemically, and biologically compatible, is known to have medical activities such as being antidiabetic, antimicrobial, antioxidant, and antitumor^70^. The antimicrobial activity of chitosan is due to its polycationic structure^71^. Positively charged chitosan interacts with the negatively charged components of the bacterial cell causing disruptions in normal cell metabolism^72^. It is reported in the literature that many materials coated with chitosan exhibit different levels of antimicrobial properties. Zhang et al. reported that chitosan-TiO_2_ composite materials exhibit strong antimicrobial activity against *E. coli*, *S. aureus*, *C. albicans* and *A. niger*^73^. In another study, Munteanu et al. found that chitosan-coated polyethylene surfaces provided 100% inhibition against *S. enteritidis* after 48 h of interaction, while providing 96.43% inhibition against *E. coli* ^74^.

Another important result obtained from the antimicrobial test is that chitosan-coated MAO surfaces have a greater effect against *E. coli* than *S. aureus*. This result shows that, in general, the chitosan-coated MAO surface is more effective against gram-negative than gram-positive bacteria. It was determined that the antimicrobial effectiveness of chitosan-coated MAO surfaces against *E. coli* is 1.58 times greater than against *S. aureus*. This result can be explained by the differences in the cellular structure of gram-positive and gram-negative bacteria. The fact that the gram-negative bacteria surface has more hydrophilic character compared to gram-positive bacteria makes them more susceptible to chitosan^75^. This higher hydrophilic property leads to greater interaction with chitosan and large changes in the structure and permeability of the cell membrane. These alterations result in bactericidal effects and bacterial death^76^. Similar studies have demonstrated that chitosan-coated surfaces have a higher inhibitory effect against gram-negative bacteria. Munteanu et al. examined the inhibitory effect of chitosan-coated films with two gram-negative bacteria, namely *S. enteritidis* and *E. coli*, and a gram-positive bacteria, *L. monocytogenes*, and reported high inhibition in gram-negatives^74^. Esmaeili et al. reported that chitosan-coated nanoparticles exhibited significant antimicrobial effect against gram-negative bacteria^77^. As a result, it was determined that chitosan-based MAO surfaces have high antimicrobial properties compared to the MAO surfaces and exhibit broad-spectrum activity that affects both gram-negative and gram-positive bacteria.

## Conclusions

In this work, antimicrobial and bioactive chitosan-based MAO biopolymer and bioceramic composite surfaces were fabricated on commercial pure Zr by MAO and dip-coating methods. The chitosan-based MAO surface was observed to be nonporous and crack-free post-fabrication dip coating, while the MAO surface was porous and rough due to the existence of micro-sparks during the process. All elements such as C, Zr, Ca, P, and O, which contributed to form antimicrobial, bioactive, and biocompatible phases, were homogeneously separated during the surfaces. The chitosan-based MAO surface indicated hydrophobic character with respect to the MAO surface because the chemical composition was changed, and the porous surface was eliminated. In vitro prediction of bioactivity and the apatite-forming abilities of the chitosan-based MAO surfaces were considerably improved compared to the plain MAO surfaces. Furthermore, bacterial adhesion to the chitosan-based MAO surfaces was less than that of plain MAO surfaces for *E. coli* and *S. aureus*.
